# Deep Convolutional Neural Networks Detect no Morphological Differences Between Culture-Positive and Culture-Negative Infectious Keratitis Images

**DOI:** 10.1167/tvst.12.1.12

**Published:** 2023-01-06

**Authors:** Kaitlin Kogachi, Prajna Lalitha, N. Venkatesh Prajna, Rameshkumar Gunasekaran, Jeremy D. Keenan, J. Peter Campbell, Xubo Song, Travis K. Redd

**Affiliations:** 1 Casey Eye Institute, Oregon Health & Science University, Portland, OR, USA; 2 Aravind Eye Hospital, Madurai, Tamil Nadu, India; 3 Francis I. Proctor Foundation, University of California San Francisco, San Francisco, CA, USA; 4 Department of Medical Informatics and Clinical Epidemiology and Program of Computer Science and Electrical Engineering, Oregon Health & Science University, Portland, OR, USA

**Keywords:** convolutional neural network, infectious keratitis, corneal ulcer

## Abstract

**Purpose:**

To determine whether convolutional neural networks can detect morphological differences between images of microbiologically positive and negative corneal ulcers.

**Methods:**

A cross-sectional comparison of prospectively collected data consisting of bacterial and fungal cultures and smears from eyes with acute infectious keratitis at Aravind Eye Hospital. Two convolutional neural network architectures (DenseNet and MobileNet) were trained using images obtained from handheld cameras collected from culture-positive and negative images and smear-positive and -negative images. Each architecture was trained on two image sets: (1) one with labels assigned using only culture results and (2) one using culture and smear results. The outcome measure was area under the receiver operating characteristic curve for predicting whether an ulcer would be microbiologically positive or negative.

**Results:**

There were 1970 images from 886 patients were included. None of the models were better than random chance at predicting positive microbiologic results (area under the receiver operating characteristic curve ranged from 0.49 to 0.56; all confidence intervals included 0.5).

**Conclusions:**

These two state-of-the-art deep convolutional neural network architectures could not reliably predict whether a corneal ulcer would be microbiologically positive or negative based on clinical photographs. This absence of detectable morphological differences informs the future development of computer vision models trained to predict the causative agent in infectious keratitis using corneal photography.

**Translational Relevance:**

These deep learning models were not able to identify morphological differences between microbiologically positive and negative corneal ulcers. This finding suggests that similar artificial intelligence models trained to identify the causative pathogen using only microbiologically positive cases may have potential to generalize well, including to cases with falsely negative microbiologic testing.

## Introduction

Corneal opacification remains a leading cause of blindness worldwide, with infectious etiologies comprising a high proportion.^1^^–^^4^ The current gold standard methods to determine the causative pathogen consist primarily of microbiologic testing, including cultures and smears (Gram stain in the case of bacterial keratitis, and potassium hydroxide [KOH] smears in the case of filamentous fungal keratitis). Unfortunately, standard microbiology tests have poor sensitivity, with false negatives occurring in up to one-half of cases. Further, cultures can take days or weeks to provide results.^5^^–^^7^ The resulting delay in directed antimicrobial therapy can lead to worse visual outcomes and an increased need for surgical intervention.

A potential alternative method for point-of-care determination of the underlying pathogen in infectious keratitis is image-based classification with deep learning, which has demonstrated promise in several preliminary studies.^8^^–^^11^ Models trained specifically to perform image-based differentiation of bacterial and fungal keratitis (the two most common etiologies of corneal ulcers) have achieved superhuman performance. Despite the promise of these early attempts to apply deep learning to this problem, these models have been trained and validated only on relatively small numbers of exclusively microbiologically positive, monomicrobial cases of infectious keratitis. Before implementation, we must validate deep learning model performance on real world, prospectively collected datasets containing bacterial, fungal, culture/smear negative, and polymicrobial corneal ulcers. However, supervised learning requires accurate gold standard labels for model training. These models interpret the morphology present in an external photograph to make the determination of the underlying cause of infection. If there are inherent morphological differences between these microbiologically positive cases and their microbiologically negative counterparts, we will introduce bias into the model by training only on the former. If there are no detectable morphological differences, then training a model only on microbiologically positive cases should not impair the model's potential for generalizability to microbiologically negative cases. Several ulcer characteristics have been theorized to be associated with culture positivity, including the type of organism, ulcer severity, history of contact lens use, and prior treatment.^12^^,^^13^ Herein, we aimed to determine whether there are morphological differences between microbiologically positive and negative ulcers that can be detected by modern deep convolutional neural networks (CNNs) to address this question.

## Methods

All adults presenting to the Aravind Eye Hospital in Madurai, India, with a clinical diagnosis of infectious keratitis were screened for potential participation in this study. After obtaining consent, eligible subjects underwent standard evaluation for corneal ulcer patients, including a detailed clinical history and slit lamp examination. All subjects then underwent external photography using a handheld Nikon D7100 camera with a 105-mm macro lens according to a standardized photography protocol, which has been successfully employed in several previous clinical trials.^14^^–^^16^

Subjects then underwent corneal scraping with heat sterilized Kimura spatulas according to the standard of care. Specimens were prepared for gram stain, KOH smear, bacterial culture on blood agar, and fungal culture on potato dextrose agar. Smear and culture results were determined by experienced microbiology technicians.

This study adhered to the tenets of the Declaration of Helsinki and was approved by the internal review board at Oregon Health & Science University and Aravind Eye Hospital (project code RES2020050BAS). Informed consent was obtained from all participants. All cases of clinically diagnosed infectious keratitis were considered eligible for inclusion, as long as the external photographs and microbiologic specimens were obtained.

Images were labeled according to their microbiologic results. Overall, both smears (Gram stain and KOH)^17^^,^^18^ and cultures^17^^,^^19^ have highly variable sensitivity and specificity, particularly for cases of bacterial infectious keratitis,^20^ with prior recommendations advocating for a combination of smears and cultures to guide clinical treatment of infectious keratitis.^21^ To address the variability in smear and culture results, we elected to establish two methods of label assignment in this study. The first (and more restrictive) method included only cases with positive cultures (bacterial, fungal, or both) in the microbiologically positive group. This approach conferred the greatest specificity for identifying culture positive cases. In the second (less restrictive) approach, cases with any positive microbiologic result (KOH smear, Gram stain, bacterial culture, or fungal culture) were considered microbiologically positive. This approach conferred high sensitivity but relatively lower specificity for label assignment. Both approaches were used to train and evaluate distinct CNNs.

Subjects were allocated randomly into the training set (80%), validation set (10%), or test set (10%). Care was taken to ensure that all images associated with a particular subject were allocated to the same image set, to prevent label leakage. During training, image augmentation was performed to minimize overfitting using random flipping along the vertical axis and random rotation up to 20° in either direction. Images were then resized according to the standard input size for these deep CNNs (224 × 224 pixels) using the bilinear interpolation algorithm. Two model architectures were evaluated: MobileNetV2 and DenseNet201. These two architectures were previously found to have the highest performance among deep CNNs for distinguishing bacterial and fungal keratitis using this same imaging modality.^8^ Each was pretrained on ImageNet, then repurposed to our application using transfer learning.^22^ The top layer of each model was replaced with a global average pooling layer followed by a single densely connected node with a sigmoid activation function, providing the model's estimated probability that an input image would be microbiologically positive or negative. Model optimization was performed using minibatch gradient descent according to the RMSprop algorithm to minimize the binary cross-entropy loss function. The loss function was weighted to account for the class imbalance present in the dataset. All model training was conducted in Python 3 using Tensorflow 2 with Keras on an AWS EC2 remote instance (Amazon Inc., Seattle, WA) with a Tesla V100 GPU (NVIDIA Corp., Santa Clara, CA). All source code is publicly available on GitHub (https://github.com/tkredd2/MobileNet_to_predict_corneal_ulcer_culture_positivity), and all hyperparameters used to train the models are available in Supplementary Table S1. A total of four deep CNNs were developed: one MobileNet and one DenseNet trained on the image set with labels assigned according to culture status only, as well as one MobileNet and one DenseNet trained on the image set with labels assigned according to both culture and smear status.

## Results

**Figure 1.**
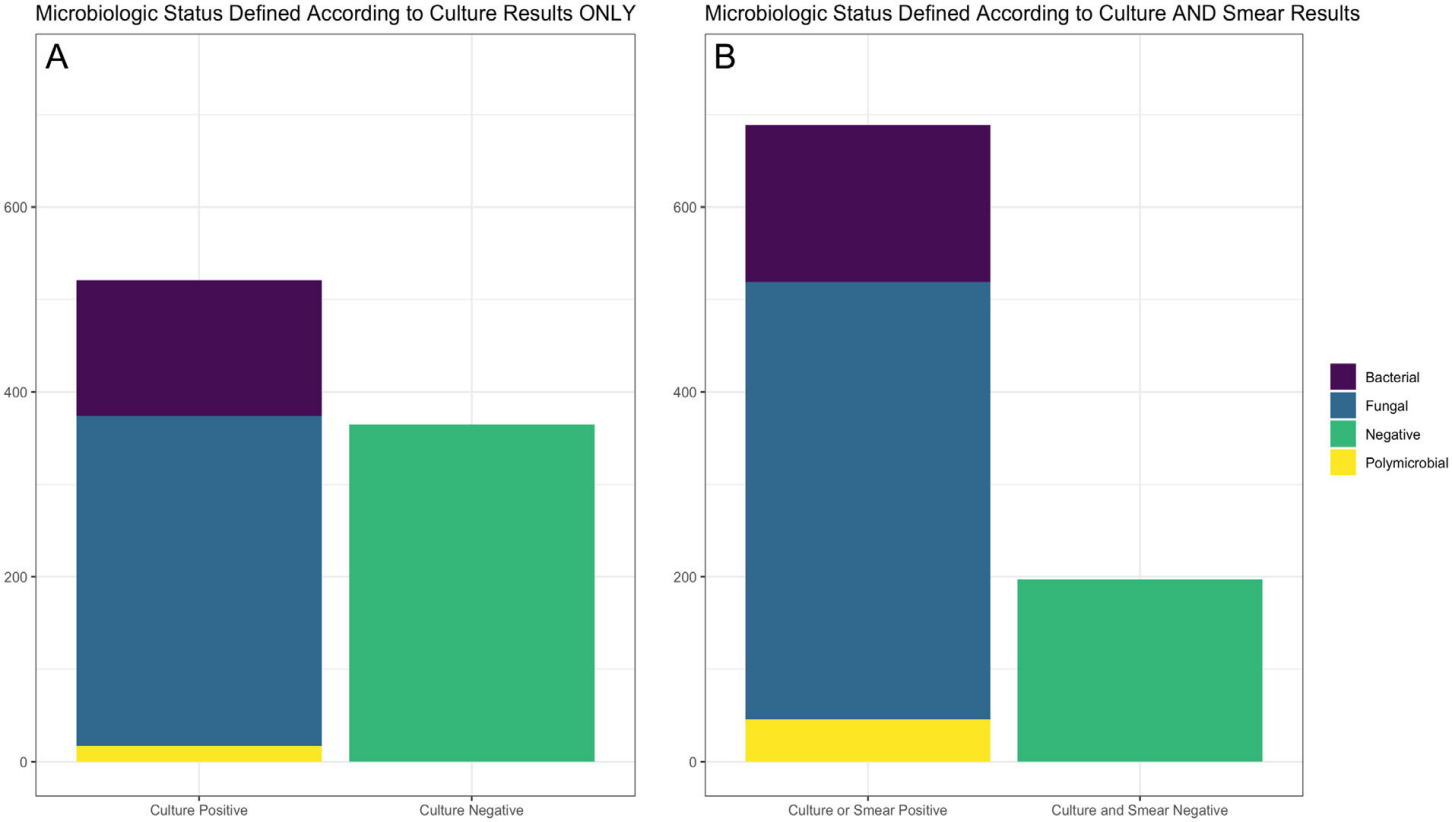
Stacked bar charts depicting the prevalence of microbiologic positivity of corneal ulcer cases in this cohort. “Microbiologic positivity” is defined according to culture results only (A) and according to culture and smear results (B). Microbiologically positive cases are further subdivided into bacterial, fungal, and polymicrobial cases.

There were 934 subjects with clinically diagnosed infectious keratitis recruited for this study. Forty-eight subjects were excluded from the analysis because of missing results on one or more microbiologic tests, resulting in 1970 images from 886 subjects in the final dataset. This included 444 ulcers of the right eye (50.2%) and 442 of the left eye (49.8%). There were 521 cases that were culture positive (59%) and 365 that were culture negative (41%). Among those that were culture positive, 164 demonstrated bacterial growth (32%), 374 demonstrated fungal growth (72%), and 17 (3%) grew both bacteria and fungi (i.e., polymicrobial). Figure 1 depicts the relative proportions of these groups. The test set defined according to culture status alone consisted of 203 images; 62 (31%) culture negative and 141 (70%) culture positive. The validation set consisted of 189 images; 57 (30%) culture negative and 132 (70%) culture positive. The training set consisted of 1578 images; 496 (31%) culture negative and 1082 (69%) culture positive.

Using the less restrictive definition of microbiologic positivity incorporating both culture and smear results, we found that 690 cases were culture and/or smear positive (78%) and 197 were culture and smear negative (22%). Among those that had positive results on either culture, gram stain, or KOH smear, 216 cases demonstrated the presence of bacteria (31%), 519 demonstrated fungi (75%), and 46 were polymicrobial (7%). The test set defined according to both culture and smear results consisted of 211 images; 28 (13%) culture and smear negative and 183 (87%) culture or smear positive. The validation set consisted of 200 images; 24 (12%) culture and smear negative and 176 (88%) culture or smear positive. The training set consisted of 1590 images; 176 (11%) culture and smear negative and 1414 (89%) culture or smear positive.

Gram stains demonstrated bacteria in 166 cases (19%), with the majority demonstrating gram-positive cocci or gram-negative bacilli (Table 1). There were 487 cases that demonstrated filamentous fungi on KOH smear (55%). There were 164 cases (19%) that demonstrated bacterial growth in culture (Table 2). The most common bacterial species was *Pseudomonas aeruginosa* (34%), followed by *Streptococcus pneumoniae* (15%) and *Nocardia* species (11%). There were 374 cases (42%) that demonstrated fungal growth in culture. *Fusarium* was the most commonly identified fungal pathogen (28%), followed by *Aspergillus* (18%) and dematiaceous molds (18%).

**Table 1.** Summary of Gram Stain and KOH Smear Findings

	*n* (%)
Gram-stain positive for bacteria	166 (19)
Gram-positive cocci	80 (48)
Gram-positive bacilli	34 (21)
Gram-negative cocci	1 (1)
Gram-negative bacilli	65 (39)
KOH or Gram stain positive for fungus	487 (55)

*Group percentages may sum to >100% owing to the presence of multiple organisms in some cases.

**Table 2.** Distribution of Pathogens Identified From Cultures

	*n* (%)
Bacterial culture positive	164 (19)
*Pseudomonas aeruginosa*	56 (34)
*Streptococcus pneumoniae*	24 (15)
*Nocardia* species	18 (11)
Other gram-negative organisms	15 (9)
*Staphylococcus aureus*	13 (8)
*Streptococcus viridans* group	12 (7)
*Corynebacteria* species	10 (6)
Other Gram positive organisms	8 (5)
*Staphylococcus* (coagulase negative)	8 (5)
*Enterobacter* species	2 (1)
Other *Pseudomonas* species (not *aeruginosa*)	2 (1)
Fungal culture positive	374 (42)
Hyaline molds	182 (49)
*Fusarium* species	104 (57)
*Aspergillus* species	66 (36)
Other/unidentified hyaline mold	12 (7)
Dematiaceous molds	66 (18)
Other/unidentified dematiaceous mold	19 (29)
*Curvalaria* species	18 (27)
*Lasiodiplodia* species	16 (24)
*Bipolaris* species	6 (9)
*Alternaria* species	4 (6)
*Exserohilum* species	3 (5)
Filamentous fungus not otherwise specified	126 (34)

**Figure 2.**
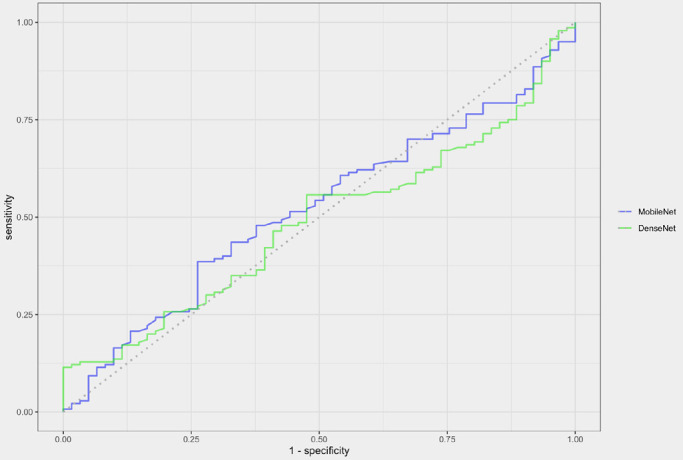
CNN performance on image set with labels defined according to culture results only. Receiver operating characteristic curves for MobileNet and DenseNet CNNs on a prospectively collected image set from a single center (1970 images total). The AUC was similar for both MobileNet (0.52) and DenseNet (0.48).

**Figure 3.**
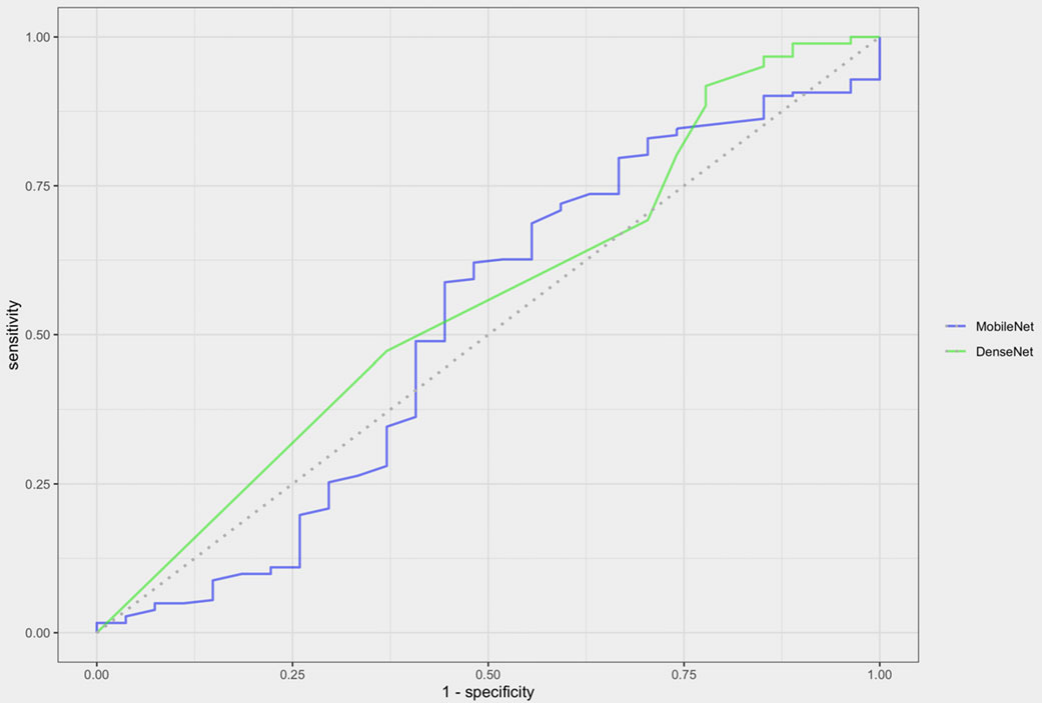
CNN performance on image set with labels defined according to culture and smear results. Receiver operating characteristic curves for MobileNet and DenseNet CNNs on a prospectively collected image set from a single center (1970 images total). The AUC was similar for both MobileNet (0.51) and DenseNet (0.56).

The area under the receiving operating characteristic curve (AUC) for differentiating microbiologically positive and negative corneal ulcers was determined for each of the four CNNs. The AUC for the DenseNet trained using only culture results was 0.48 (95% confidence interval [CI], 0.40–0.57) (Fig. 2). The AUC for the MobileNet trained using only culture results was slightly higher at 0.52 (95% CI, 0.44–0.60). The AUC for the DenseNet trained using culture and smear results was 0.56 (95% CI, 0.44–0.67) (Fig. 3) and the AUC for the MobileNet trained using culture and smear results was 0.51 (95% CI, 0.38–0.65).

## Discussion

These results demonstrate that two different CNN model architectures did not identify morphological differences between clinical photographs of microbiologically positive and negative corneal ulcers, regardless of how microbiologic positivity is defined. This work has implications for the future development of diagnostic artificial intelligence models for infectious keratitis.

Prior studies have demonstrated that these computer vision model architectures are capable of recognizing meaningful morphological differences between bacterial and fungal keratitis in corneal photographs.^8^^–^^11^ In this study, the two CNN architectures we tested were not able to identify significant morphological differences between microbiologically positive and negative cases of infectious keratitis. This lack of distinguishing morphological characteristics may suggest that training a model to determine the underlying cause of infection (i.e., bacterial vs. fungal) using only microbiologically positive cases should not impair the model's potential for generalizability to microbiologically negative cases. Based on this finding, we suggest future deep learning models for this application should continue to be trained using supervised learning with cases possessing a reliable label (i.e., microbiologically positive corneal ulcers), and evaluated on population-based samples including microbiologically positive and negative cases using techniques such as latent class analysis, which enable estimation of the sensitivity and specificity of a diagnostic test, even in the absence of a gold standard.^23^

The epidemiology of the etiologies for infectious keratitis included in this study is comparable to prior studies in South India. Lalitha et al.^7^ (2015) examined the trends of bacterial and fungal keratitis in South India over a 10-year period from 2002 to 2012 using smears as the main outcome. They reported that 61.7% of the documented smears revealed an etiologic organism, compared with our reported culture positivity of 59.0%. Based on our smear results, we had a slightly higher proportion of fungal-positive smears (55%) compared with their 34% and slightly lower proportion of bacterial-positive smears (19.0%) compared with their 24.7%. The most common bacterial species (*Streptococcus pneumoniae, Pseudomonas aeruginosa,* and *Nocardia)* and fungal species (*Fusarium* and *Aspergillus*) were the same between our study and this prior work.^7^ Other studies assessing the epidemiology of bacterial and fungal keratitis in South India have also reported similar proportions of culture positivity and most common etiologic subspecies.^24^^,^^25^ However, the epidemiology of corneal ulcers demonstrates significant regional variability; thus, these results may not be generalizable particularly to nontropical regions of the world where fungal keratitis is less prevalent.

Several potential limitations should be considered. First, the term culture-negative referred to images that did not have positive bacterial or fungal results, but it is possible that a few of these culture-negative cases included other infectious etiologies (e.g., *Acanthamoeba* or viral keratitis) or sterile keratitis (e.g., interstitial keratitis, peripheral ulcerative keratitis, etc.). However, all subjects with a suspected acanthamoeba-related ulcer undergo confocal microscopy at Aravind, which would exclude a large majority of potential cases. In addition, viral necrotizing keratitis is very rare and would be less likely to masquerade as a culture-negative case, and the clinical volume of infectious keratitis in South India is such that the diagnostic expertise of these cornea specialists is very high.^7^ Second, variable image quality likely impacts model performance. It is possible that subtle differences in morphology and appearance may not be detected if image quality is poor, which could explain our findings. Similarly, it is possible that morphological differences do exist between microbiologically positive and negative ulcers, but these CNNs simply do not have sufficient representational capacity to recognize these differences using this imaging modality. However, these same CNN architectures were capable of accurately distinguishing between bacterial and fungal etiologies using this same image set, suggesting that image quality and the CNNs themselves are sufficient to reliably identify even subtle differences in ulcer morphology from corneal photographs.

Our results demonstrate that these state-of-the-art computer vision models were not able to differentiate between microbiologically positive and negative corneal ulcers based on external photographs, regardless of whether cultures or smears were used to determine the microbiologic status. These findings suggest that the CNNs used to distinguish the underlying etiology of infection in subjects with corneal ulcers may generalize well to microbiologically negative cases, even if they are trained using supervised learning with only microbiologically positive cases. Additional model evaluation studies in population-based samples are a necessary next step in the determination of whether these deep learning models may provide clinical benefit to patients with this potentially blinding condition.

## Supplementary Material

Supplement 1
