# Supplementary material for: Deep Convolutional Neural Networks Detect no Morphological Differences Between Culture-Positive and Culture-Negative Infectious Keratitis Images
Source: Transl Vis Sci Technol. 2023 Jan 6;12(1):12. doi: 10.1167/tvst.12.1.12 (PMC9836011; doi:10.1167/tvst.12.1.12)
Supplement: Supplement 1 [file tvst-12-1-12_s001.docx]

Supplementary Table S1. Hyperparameters for Training Models

|  | | |  | **Feature Extractor*** | | | **Fine Tuning**† | | | |
| --- | --- | --- | --- | --- | --- | --- | --- | --- | --- | --- |
|  | | | Batch Size | Epochs | LR | Dropout Rate | Epochs | LR | Dropout Rate | Last Frozen Layer |
| Image Set 1‡ | | |  | | | | | | | |
| MobileNet | | 64 | 10 | 1e^-3^ | 0.3 | 100 | 1e^-4^ | 0.5 | 98 |  |
| DenseNet | | 64 | 10 | 1e^-4^ | 0.3 | 20 | 1e^-5^ | 0.3 | 600 |  |
| Image Set 2§ | | |  | | | | | | | |
| MobileNet | 64 | 10 | 1e^-4^ | 0.3 | 100 | 1e^-4^ | 0.5 | 143 |  |  |
| DenseNet | 64 | 10 | 1e^-4^ | 0.3 | 80 | 3e^-3^ | 0.3 | 400 |  |  |
| * “Feature Extractor” phase of training denotes freezing of all layers except the top layer during training. This is done prior to “Fine Tuning” to reduce propagation of high loss through the earlier layers.  † “Fine Tuning” phase of denotes additional training after the “Feature Extractor” phase wherein a defined number of the deepest layers (indicated by “Last Frozen Layer”) are unfrozen during training, to learn task-specific feature representations.  ‡ “Image Set 1” indicates image set with labels defined according to culture status *only*  § “Image Set 2” indicates image set with labels defined according to culture *and smear* status  Abbreviations: LR = learning rate | | | | | | | | | | |
